# PEGylation-Driven Remodeling of the Protein Corona on PLGA Nanoparticles: Implications for Macrophage Recognition

**DOI:** 10.1021/acs.biomac.5c01369

**Published:** 2025-10-27

**Authors:** Lucio Spinelli, Pasquale D’Anna, Elva Morretta, Chiara Cassiano, Virgilio Piccolo, Martina De Rosa, Rebecca Amico, Paola De Cicco, Diego Brancaccio, Claudia Conte, Angela Zampella, Fabiana Quaglia, Maria Chiara Monti

**Affiliations:** 9307 University of Napoli Federico II, Department of Pharmacy, Via D. Montesano 49, Naples 80131, Italy

## Abstract

The formation of a Protein Corona (PC) on the surface of nanoparticles (NPs) is a critical event that shapes their biological identity and governs interactions with the immune system. In this study, we investigated the composition of the PC formed on mixtures of PLGA and PEG–PLGA NPs, aiming to elucidate the link between NPsurface chemistry, proteomic fingerprint in cell culture medium, and uptake by bone marrow-derived macrophages (BMDMs). NPs showed different sizes but comparable actual PEG amount exposed on the surface, which is significantly lower than the theoretical values. The PC, isolated using a standardized microfiltration protocol, revealed distinct patterns of protein adsorption as a function of the PEG density. Uptake studies in BMDMs revealed a strong inverse relationship between PEG surface density, PC composition, and macrophage internalization, supporting the hypothesis that the opsonin/dysopsonin balance is more critical than a single protein interaction. In conclusion, this work demonstrates that the PEG surface density is not the only determinant of PC composition. These findings underscore the importance of rigorous surface characterization and PC profiling to predict and tune nanocarrier performance *in vivo*.

## Graphical abstract

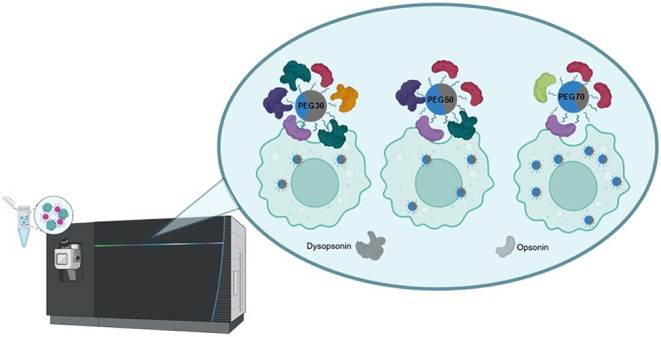

## Introduction

Nanotechnologies are emerging as transformative tools for the safe and effective delivery of a wide range of therapeutic agents, owing to their distinctive capacity to preferentially target specific tissues and organs.^1^ Upon contact with biological fluids, nanoparticles (NPs) spontaneously interact with biomacromolecules—predominantly proteins—leading to the formation of a dynamic biomolecular layer known as the protein corona (PC). The selective enrichment of specific proteins on the NP surface confers a new “biological identity” to the particles, which significantly influences their interaction with target cells, particularly immune cells.^2^ Among immune cells, monocytes and their differentiated progeny, macrophages, play a pivotal role in regulating NPs fate. Monocytes migrate to tissues upon inflammatory stimuli, where they differentiate into macrophages. These tissue-resident macrophages are central to NP recognition, uptake, and clearance.

In the context of nanomedicine, the interaction between macrophages and NPs represents a double-edged sword: on one hand, it constitutes a major biological barrier, leading to rapid sequestration and clearance of nanocarriers from circulation; on the other hand, it offers a unique opportunity to exploit macrophages as therapeutic targets, particularly in inflammatory, infectious, or tumor-associated microenvironments.^3^

The physicochemical properties of NPs—namely, their size, surface charge, and surface chemistry—play a critical role in dictating the composition, abundance, and conformational profile of the adsorbed proteins, collectively referred to as the PC fingerprint. A thorough understanding of the relationship between the *chemical identity* (intrinsic material characteristics) and the *biological identity* (PC composition) of NPs is, thus, essential for the rational design of novel nanosystems with tailored immune recognition profiles and controlled pharmacokinetics.^4,5^

Among the various classes of biodegradable nanocarriers, amphiphilic polyethylenglycol-polylactide-co-glycolide acid (PEG–PLGA) NPs represent a paradigmatic case, wherein surface chemistry critically modulates biological interactions. These NPs are engineered to feature a hydrophilic PEG corona that enhances colloidal stability and prolongs circulation time following intravenous administration.^6^ Even in*in vitro* conditions, such as incubation with fetal bovine serum (FBS), NPs acquire a biological identity through protein adsorption, which significantly influences cellular uptake, trafficking, and toxicity profiles.^7–10^

The surface density of PEG chains determines their spatial conformation on the NP surface, typically classified as mushroom or brush regimes that in turn affect protein binding, particularly by opsonins. Despite its importance, PEG surface density is not routinely quantified, making the relationship among NP surface chemistry, PC composition, and the resulting biological effects difficult to elucidate. Notably, only a limited number of studies have systematically examined how varying degrees of PEGylation influence the PC of PEG–PLGA NPs and how such variations correlate with immune recognition and cellular uptake.^11–15^ This is further complicated by the often unverified assumption that PEG is fully and uniformly displayed on the NP surface—a notion contradicted by surface characterization data in several studies.^16,17^

Methodological considerations are another important but frequently overlooked component of PC research. While advances in mass spectrometry-based proteomics have made it possible to perform detailed and high-resolution analyses of PC composition, the reliability of these data critically depends on the methods used to isolate NPs bearing the PC.^18^ Inappropriate or overly harsh isolation procedures can disrupt the native PC structure, leading to biased or incomplete proteomic profiles and potentially misinforming subsequent biological interpretations.^19,20^ The most adopted approach, centrifugation followed by resuspension, has been shown to perturb the nano–bio interface by disrupting spontaneous agglomerates formed during incubation.^19–21^ These limitations underscore the urgent need for isolation methods capable of preserving the *in situ* structure and composition of the PC.

In this study, we developed and systematically characterized a series of PEG–PLGA NPsdiffering in PEG content, with particular emphasis on their surface physicochemical properties. We then compared three distinct methods for PC isolation and identified the most suitable approach^22^ for recovering the corona formed in the presence of FBS, a commonly used protein source in *in vitro* cell culture. Proteomic profiling of the isolated coronas revealed both shared and formulation-specific protein signatures, including proteins known to mediate immune recognition. Finally, we investigated the impact of these PC fingerprints on NPuptake by murine-derived macrophages, establishing a clear link between the PEGylation degree, biological identity, and cellular interactions of PLGA-based nanocarriers.

## Experimental Section

### Materials

Poly­(lactide-*co*-glycolide) (PLGA, Resomer RG 502 H acid terminated, viscosity 0.16–0.24 dL/g, Mn = 7,000–17,000 Da) was purchased by Evonik Industries, while methoxy-poly­(ethylene glycol)-*b*-poly­(lactide-*co*-glycolide) (mPEG–PLGA, Mn = 5655 Da with PEG 2 kDa, PI = 1.22, LA:GA 50:50) was provided by PolySciTech, Akina, Inc. Acetone, Tetrahydrofuran (THF), and Dichloromethane (DCM) were purchased from VWR International S.r.l. 1,1′-Dioctadecyl-3,3,3′,3′-tetramethylindocarbocyanine perchlorate (DiI) was provided by Thermo Life Science. FBS and phosphate-buffered saline were purchased from Euroclone, Sepharose CL-2B, β-mercaptoethanol, and Microcon centrifugal filters (300 kDa MWCO, 0.5 mL) were from Sigma-Aldrich; Coomassie Brilliant Blue 5 and Laemmli buffer were from BioRad. Solvents and formic acid for LC-MS were purchased from DelTek, and an EASY-Spray PepMAP Neo C18 column was from Thermo Fisher Scientific. Trypsin/Lys-C Mix, Mass Spec grade, was from Promega.

### Nanoparticle Preparation

NPs were made of 100% PLGA­(PLGA100) or of PLGA/PEG_2k_–PLGA_2k_ mixtures at three different mass ratios (7:3, 5:5, and 3:7), named PEG30, PEG50, and PEG70, respectively, to achieve different PEGylation degrees. An organic phase (OP), constituted by a water-soluble, volatile organic solvent solubilizing the polymer and the fluorescent cargo, was nanoprecipitated in water (WP) at an OP/WP ratio of 1:2 v/v without the addition of surfactants. Briefly, the OP was prepared by dissolving 10 mg of PLGA or PLGA PEG_2k_–PLGA_2k_ at different mass ratios (7:3, 5:5, and 3:7) in 1.95 mL of acetone and adding 50 μL of the fluorescent probe stock of DiI in THF (1 mg/mL). The OP was added at a flow rate of 1 mL/min to 4 mL of stirring water by using a syringe pump (Aladdin SyringeONE Programmable Syringe Pump, WPI Inc.). The mixture was left under stirring for 30 min at rt, and then the organic solvent was evaporated through a rotary evaporator (120 rpm). Acetone evaporation was completed in 8 min.

The encapsulation efficiency of the fluorescent dye was assessed by UV–vis measurements on a GloMax Discover microplate reader (Promega Inc.) through an indirect method. Briefly, a calibration curve of DiI in THF was generated at a concentration of 1 mg/mL, followed by serial dilutions. The freshly prepared NPs were centrifuged at 20,000 × *g* for 30 min, and the supernatants were collected and the absorbance measured at a wavelength of 550 nm. The THF was used as the background control.

### Nanoparticle Characterization

The hydrodynamic diameter (*D*_H_), polydispersity index (PDI), and zeta potential (ζ) of NPs were determined on a Zetasizer Ultra (Malvern Instruments Ltd., UK). In particular, *D*_H_ was represented as the light intensity scattered by the NPs(%). The measurements were taken on freshly prepared NPs and following their incubation with FBS and PC isolation. Each determination is the mean of three independent experiments. Fixed aqueous layer thickness (FALT) measurements were carried out by monitoring the influence of ionic strength on the particle surface. Different amounts of NaCl stock solutions at different concentrations were added to an NP dispersion in water (2.5 mg/mL), and the ζ potential of the samples was measured. A plot of ln­(ζ) against 3.33·[NaCl]^0.5^ results in a straight line, where the slope represents the thickness of the PEG shell in nm.

### Quantification of Surface PEG

The PEG surface density was calculated according to the following formula:

1 $$PEG\;surface\;density=\frac{\left(exposed\;PEG\;wt\%\right)\times V\times \rho \times N_{A}}{\left(PEG\;MW\right)\times S}$$

Where *V*, ρ, and *S* are the volume, the density, and the surface area of NPs, wt % is the average mol, and *N*_A_ is Avogadro’s number. *V* and *S* were estimated from *D*_H_, and the ρ of NPs was assumed to be 1.2 g cm^–3^.

The theoretical exposed PEG wt % was calculated assuming that PEG was completely exposed on the NP surface. The actual exposed PEG wt % was evaluated by ^1^H NMR. Spectra were recorded for either NP dispersed in D_2_O or dissolved in CDCl_3_ at ∼2.5 mg/mL, using a Bruker AVANCE NEO 600 MHz spectrometer equipped with a *z*-gradient 5 mm triple-resonance probe head. Measurements were carried out at 298 K in 5 mm NMR tubes with a sample volume of 600 μL. The NOESYGPPR1D pulse sequence was employed, acquiring 32 scans. The amount of PEG was calculated by comparing the integral of the −CH_2_– resonance at 3.6 ppm of mPEG in D_2_O with the corresponding signal in CDCl_3_.

Conformation of PEG on the NP surface was calculated from the ratio between the Flory radius (*R*_*f*_) and the average distance between adjacent PEG chains (*d*):

2 $$d\approx \sqrt{\frac{1}{\sigma }}$$

where σ is the PEG grafting density (chains/nm^2^). *R*_*f*_ was calculated according to:

3 $$R_{f}\approx a\cdot N^{3/5}$$

where *a* is the monomer length and *N* is the number of monomers in the chain. For PEG 2kDa, *a* is ≈0.35 nm, and *N* is ≈45, resulting in a *R*_*f*_ ≈ 3.46 nm.

### Isolation of PC-Coated NPs

PLGA NPs and PLGA/PEG_2k_–PLGA_2k_ NPs tagged with DiI (40 μL at a final concentration of 500 μg/mL) were incubated with 10% FBS (160 μL) for 1 h at 37 °C under constant and gentle shaking to induce PC formation. FBS was diluted at 10% using 1× PBS. As a negative control, NPs were replaced with the same volume of phosphate-buffered saline (PBS) (NaCl 137 mM, KCl 2.7 mM, NaH_2_PO_4_ 10 mM, K_2_HPO_4_ 1.8 mM, pH 7.4). An additional control of only PLGA NPs in PBS was used for recovery monitoring during isolation. The incubation was repeated in triplicate.

Three methods to isolate PC-coated NPs were tested by using DiI-loaded PLGA NPs as a representative NP sample and then applied to DiI-NPs of PLGA/PEG_2k_–PLGA_2k_.

#### Centrifugation

200 μL of NPs were incubated in FBS and then centrifuged at 21,000 *g* for 15 min. Then, the supernatant was discarded, and the pellet was washed with 200 μL of PBS and centrifuged at 21,000 *g* three times. The pellet was resuspended in 500 μL of PBS. A suitable control of the FBSalone at 10% was also used.

#### Size Exclusion Chromatography

200 μL of NPs previously incubated with FBS were loaded on 8 mL column packed with Sepharose CL-2B. PBS was used as the eluent, and 10 fractions of 500 μL each were collected for each chromatographic run and then lyophilized. A suitable control of the FBSalone at 10% was also used.

#### Microfiltration

200 μL of NPs were incubated in FBSand were loaded on Microcon centrifugal filters (300 kDa MWCO, 0.5 mL). A suitable control of FBS at 10% without NPs was also used and treated in parallel. Each sample was further diluted with PBS to a total volume of 500 μL and centrifuged at 5000 *g* for 20 min at 4 °C, for five times. After each centrifugation step, the permeate was collected and its volume was measured. Before the following centrifugation step, an equal amount of PBS was added on top of the filter and the volume was diluted back to 500 μL. Subsequently, the filter unit was reversed and an additional centrifugation step, at 1000 *g* for 3 min, was carried out to collect the PC-covered NPs.

The last method was considered to be the most reliable and was employed for PEGylated NPs.

### PC Characterization

#### Proteins and NPs Quantification

PC-coated NPs (80 μL) were tested by Bradford Assay to assess the amount of adsorbed proteins, using bovine serum albumin (BSA) at concentrations ranging from 0.156 to 5 μg/μL to build the calibration curve. The samples were loaded in a 96-multiwell at final volume of 200 μL and incubated shortly, and the absorbance was read at 595 nm on the VICTOR Nivo Multimode Microplate Reader (Revvity).

The NP concentration was assessed by measuring the fluorescence of DiI (λex = 530 nm, λem = 570 nm) using 25 μL of each sample on the VICTOR Nivo Multimode Microplate Reader (Revvity).

#### SDS-PAGE

PC-coated PLGA NP samples were freeze-dried, if needed, and subsequently redispersed in 20 μL of 1X Laemmli buffer with 2% β-mercaptoethanol. Proteins were boiled at 95 °C for 5 min, loaded on a 12% polyacrylamide gel, and separated maintaining a constant voltage of 120 V. A 10 μg portion of 10% FBS was also used as a positive control. Coomassie Brilliant Blue 5 staining solution (1:1 dilution) was used to dye the proteins.

#### PC Digestion, NanoLC-MSMS, and Data Analysis

PC-coated NPs PEG30, PEG50, PEG70, and PLGA100 were digested using the Filter-Aided Sample Preparation (FASP) protocol, as reported by Wíniewski.^23^ The obtained peptide mixtures were dried under vacuum and dissolved in 0.1% formic acid (FA) for the subsequent nano LC-MS/MS analysis. 5 μL of each peptide mixture was loaded on an Orbitrap Eclipse Tribrid Mass Spectrometer (Thermo Fisher Scientific) coupled to a Vanquish Neo UHPLC System, equipped with an EASY-Spray PepMAP Neo C18 column (2 μm, 100 Å, 75 μm × 50 cm, Thermo Fisher Scientific) at a flow rate of 270 nL/min with the following gradient: 0.1 min at 3% B, 0.1 to 75.1 min to 40% B, 75.1 to 75.2 min to 99% B, then held at 99% B for 12.9 min, and re-equilibrated for 8 min at 3% B (A: H_2_O, 0.1% FA; B: 80% CH_3_CN, 20% H_2_O, 0.1% FA). The mass spectrometer was operated in data-dependent acquisition mode. Full-scan MS spectra were acquired with the scan range of 375–1500 *m*/*z*, a full-scan normalized automatic gain control (AGC) target 100% at 240,000 resolution, and a maximum injection time of 50 ms. MS^2^ spectra were generated based on cycle time (normalized collision energy of 30%), and the fragment ions were acquired in the ion trap with a normalized AGC target of 300% and a maximum injection time of 35 ms. The experiments were performed as independent triplicates, and three nano-LC-MSMS analyses were run for each sample.

Protein identification and label-free quantification were then achieved through Proteome Discoverer (version 3.1.0.638). The analysis was performed by Sequest against a reviewed*Bos taurus*database (SwissProt, March 2024, 6415 entries) with the following parameters: trypsin digestion; a maximum of two missed cleavages; cysteine carboxyamidomethylation as a fixed modification. Protein identification scoring was done using the percolator node; in the LFQ approach, each protein’s abundance was calculated by summing the sample abundance of the connected peptide groups by Proteome Discoverer. The identified and quantified proteins were visualized as a heatmap, comparing the corona against both controland FBS. A gene ontology analysis was carried out: proteins were first filtered to account only for those common to at least 66% of the experiments (6 out of the 9 biological and injection replicates) and the gene list was submitted to FunRich, a functional annotation tool (www.funrich.org). Finally, the proteins were clustered by a biological process.

The equation used for the semiquantitative assessment of the proteins enriched on the corona was described in previous studies:^24^

$$NpSpC_{k}=\left(\frac{\left(SpC/{\left(M_{w}\right)}_{k}\right)}{\overset{n}{\underset{t=1}{\sum }}\left(SpC/{\left(M_{w}\right)}_{t}\right)}\right)\times 100$$

*NpSpCk* is the normalized percentage of the spectral count for protein *k*; *SpC* is the spectral count; and *M*_*w*_ is the molecular weight (kDa) of protein *k*. The *SpC* of each identified protein was normalized to the protein mass and expressed as the semiquantitative assessment of the protein. This correction is based on the protein size and evaluates the relative contribution of each protein enriched in the corona of the NPs.

#### Cell Viability Assay

Bone marrow cells were isolated from the femur and tibia of male C57BL/6 mice and cultured in RPMI 1640 medium containing 100 U/mL penicillin/streptomycin, 1 mM HEPES (pH 7.4), and 10% FBS, supplemented with macrophage colony-stimulating factor (M-CSF) (50 ng/mL) (Miltenyi Biotec, Germany) for 6 days to induce differentiation into macrophages. All animal experiments were performed following the protocols evaluated and approved by the Italian Ministry of Health (approval number C6AC4.N.QUS).

BMDMs were seeded in 96-well plates at a density of 5 × 10^4^ cells/well and incubated with PEG30, PEG50, and PEG70 formulations at the concentration of 50 μg/mL for 24 h. Then, MTT solution (5 mg/mL) was added to each well, followed by a standard incubation period of 3 h at 37 °C. The resultant formazan crystals were solubilized with DMSO, and the absorbance was measured at 570 nm using a Multiskan GO microplate reader (Thermo Fisher Scientific, MA, USA). Percent of cell viability was determined relative to the untreated control wells.

#### Macrophage Uptake by Flow Cytometry

A quantitative assessment of the cell uptake of DiI-labeled PEG30, PEG50, and PEG70 NPs was performed by using flow cytometry. Briefly, BMDMs were seeded in 12-well plates at a density of 5 × 10^5^ cells/well and allowed to adhere overnight. Cells were then incubated with the formulations (50 μg/mL) for 1, 4, or 24 h in 10% FBS containing medium. In experiments with LPS-stimulated BMDMs, a pretreatment with LPS (1 μg/mL) was performed for 30 min prior to incubation with the formulations, and uptake was assessed after 1, 4, or 24 h in 10% FBS containing medium. After the incubation times, the cells were detached, washed in FACS buffer (PBS with 0.1% BSA), and then incubated with a fixable viability dye. Internalization of the formulations was confirmed by measuring cell-associated fluorescence by flow cytometry (BriCyte E6, Mindray, P.R. China). For each sample, 20,000 events were acquired and analyzed using FlowJo software. Cell uptake was quantified by measuring the percentage of DiI-positive cells and the mean fluorescence intensity (MFI).

#### Fluorescence Assay by Confocal Microscopy

BMDMs were seeded in a 4-well chamber slide system at a density of 2.5 × 10^5^ cells/well, incubated overnight, and treated with 50 μg/mL of DiI-labeled PEG30, PEG50, and PEG70 formulations for 1, 4, or 24 h in 10% FBS containing medium. In experiments with LPS-stimulated BMDMs, a pretreatment with LPS (1 μg/mL) was performed for 30 min prior to incubation with the formulations. After the incubation period, the cells were rinsed twice with PBS and stained with Hoechst 33342 (1 μg/mL) for 15 min prior to imaging. The cells were imaged with a Zeiss LSM 980 Airyscan-2 confocal microscope (Carl Zeiss AG, Germany).

## Results

### Properties of PEGylated PLGA NPs

NPs were prepared from PLGA and from mixtures of PLGA and PEG–PLGA at three reciprocal ratios (30:70, 50:50, and 70:30, named PEG30, PEG50, and PEG70, respectively) and at the same polymer concentration of 10 mg/mL (Figure 1A). As reported in the literature, the molecular weight of PEG chains plays a crucial role in dictating NPs behavior in the biological environment, particularly in reducing protein adsorption on the NP surface and limiting recognition by the mononuclear phagocyte system (MPS).^17^ These effects are most evident for PEG chains in the 2–5 kDa range, whereas higher molecular weights do not provide additional improvement in surface shielding.^17^ In our study, we selected PEG–PLGA with PEG chains of 2 kDa, which lies within this optimal range, with the aim of comparing a PEGylated delivery system to its non-PEGylated counterpart. NPs were prepared at a theoretical PEG grafting density between 0.89 and 1.42 PEG chains/nm^2^, resulting in *R*_*f*_ */d* > 2, a value reported to provide a dense PEG brush on the NP surface.^25^ NPs were produced by nanoprecipitation, since it is a simple and robust process compatible with continuous manufacturing. The method is particularly well-suited for scale-up through microfluidic technologies, enabling precise control over the particle size and batch-to-batch reproducibility, a key parameter for clinical translation. The polymers were dissolved in acetone, providing a low-viscosity solution that promotes polymer chain mobility and nanostructuring in core–shell NPs upon nanoprecipitation in water. The amphiphilic nature of PEG–PLGA enabled the formation of NPs without the addition of surfactants, which is an advantage, as it simplifies purification steps. NPs were loaded with the fluorescent tag DiI, which allowed for evaluation of trafficking in macrophages.

**Figure 1:**
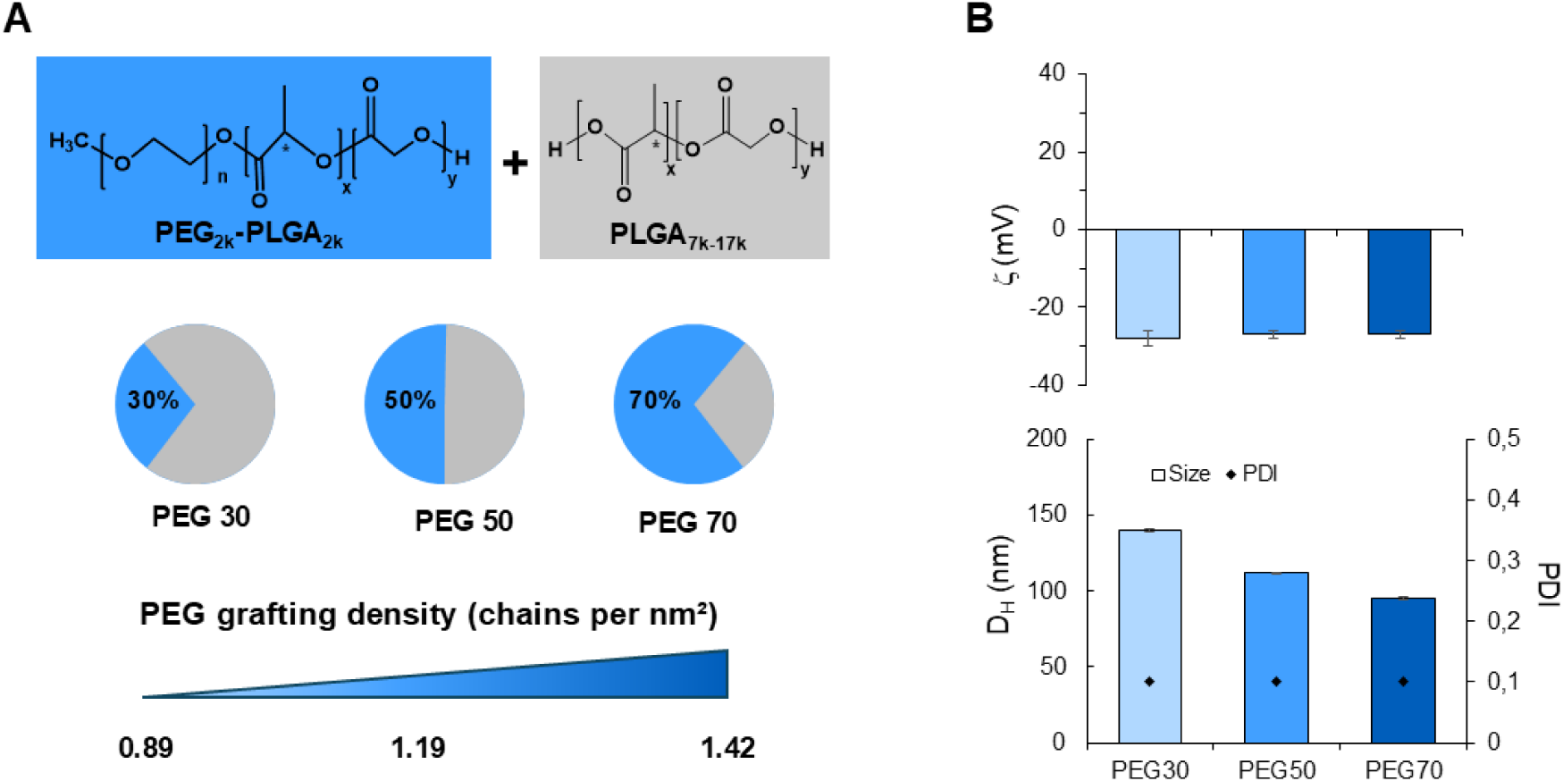
A) PEGylated NPs were prepared at 10 mg/mL using a mixture of PLGA and PEG–PLGA (30%, 50%, and 70% of the total polymer weight). The theoretical PEG grafting density of the NPs was calculated according to eq 2. B) Properties of DiI-loaded PEGylated NPs.

Hydrodynamic diameter (*D*_H_), polydispersity index (PDI), and zeta potential (ζ) of the PEGylated NPs are reported in Figure 1B. All NP variants were monodispersed (Figure S1A) with a PDI of ca. 0.1. The *D*_H_ spanned the range of 95–140 nm and progressively increased as the fraction of PEG–PLGA increased reasonably due to its amphiphilic properties and faster desolvation of the polymer chains. The surface charge of the NPs was approximately −30 mV, due to the presence of carboxylated PLGA end groups, and remained consistent across all formulations. Concerning the encapsulation efficiency, the entrapment of DiI inside the NPs was ≥75% (Figure S2).

### Surface Characterization of PEGylated NPs

The Fixed Aqueous Layer Thickness (FALT) surrounding NPs was determined by measuring their ζ potential at increasing NaCl concentrations. The increase of the ionic strength induces a decrease of ζ because of the variation of the slipping plane of the PEG fringe away from the surface of the NP core. As reported in Figure 2A, the slope value of the linear regression line yields a shell thickness ranging from 3.53 to 3.79 nm, which is coherent with the PEG *R*_*f*_ of 3.5 nm. Here, we emphasize that the overall size of PEGylated NPs is dominated by the polymer core rather than the thickness of the shell. The core is 54, 62, and 74 times larger than the NP shell for PEG30, PEG50, and PEG70 NPs, respectively. It is worth reflecting on the common graphical representation of PEGylated NPs, which often depicts long PEG chains extending far from relatively small cores, potentially oversimplifying or exaggerating the actual spatial dimensions and conformation of PEG in biological environments, leading to ambiguous interpretation.

**Figure 2:**
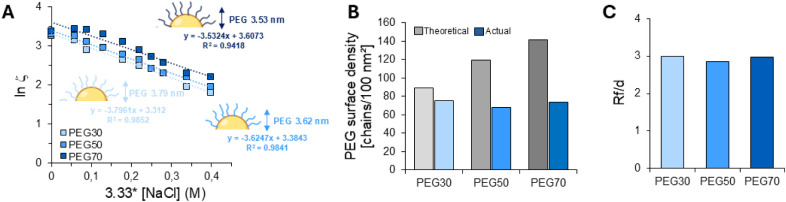
A) Plot of sodium chloride molar concentration vs ln ζ and corresponding linear regression of the data (*r* ^2^ ≥ 0.95), where the slope value corresponds to the NP shell thickness (nm). SD values are omitted for clarity purposes and are never greater than 0.15. B) PEG surface density (gray, theoretical values; blue, actual values from ^1^H NMR analysis) according to [1]. C) Conformation of PEG calculated according to [2] and [3].

Because the presence of PEG plays a crucial role in driving PC formation, quantifying the actual amount of PEG on the NP surface by robust techniques such as ^1^H NMR spectroscopy is relevant. The actual PEG amounts were approximately 84%, 57%, and 52% of the theoretical content for PEG30, PEG50, and PEG70, respectively. Notably, the PEG surface density was lower than the designed one (Figures 2B and S3), approximately half of that theoretically calculated for PEG70. Geometric considerations can explain this apparent decoupling between total PEG content and surface density: smaller NPs, such as in the PEG70 formulation, present a reduced surface area, which leads to a higher local PEG density per unit area, even when the total PEG incorporated is lower. Despite this, PEG surface densities approached the critical threshold for the brush regime of 3, suggesting a dense brush conformation (Figure 2C). Our findings are in line with previous studies^16^ and remark the relevance of assessing the actual PEGylation extent of NPs, especially when NP structure is related to biological data.^17^

### Optimization of PLGA NPs PC Isolation from FBS

To optimize the isolation procedure for PC, DiI-loaded NPs made only of PLGA were used as a reference standard, given their well-known propensity to adsorb proteins on their surface.^26^ PLGA NPs were prepared with the same procedure, and their properties are reported in Figure S1B.

DiI-loaded PLGA NPs were incubated with 10% FBS, a typical serum concentration used in *in vitro* cell culture experiments, for 1 h at 37 °C under gentle shaking. Then, the samples were treated by three different protocols to separate the PC from the surrounding matrix: (i) centrifugation-redispersion, (ii) Size Exclusion Chromatography (SEC), and (iii) microfiltration through a 300 kDa filter unit. In parallel, 10% FBS (without NPs) and NPs in PBS (without FBS) were incubated under the same conditions and used as opportune control samples.

In the centrifugation-redispersion process, the centrifugation speed and duration as well as the washing buffer and number of washings were all carefully adjusted. The optimized procedure consisted of centrifugation at 21,000 *g* for 15 min, followed by two washing steps with PBS at the same speed, and then resuspension of the PC in the initial volume of PBS.

Using the SEC method, the NPs with their PC were separated from the background proteins by using a small-bed column filled with Sepharose CL-2B resin, which was manually packed and operated at atmospheric pressure. Each sample of NPs incubated in 10% FBS was loaded onto the column and collected in ten fractions. Larger molecules were excluded from the column bead and eluted in the first fractions. Then, fluorescence and Bradford analysis were used to determine which fraction had the same maximum percentage of NPs and proteins (Fraction 4, Figure S4 Panels A and B). A lyophilization step was necessary before SDS-PAGE due to the sample dilution involved in this process.

Lastly, 300 kDa cutoff centrifugal filters were employed for the microfiltration procedure. The samples containing 10% FBS-NPs forming the PC and 10% FBS alone were loaded separately, diluted in PBS and mildly centrifuged. The procedure was repeated three times, and the final sample volume was kept unaltered. Approximately all the proteins contained in the sample of 10% FBS alone, used as a negative control, passed through the 300 kDa cutoff centrifugal filters after two extensive washes with PBS, as expected (Figure S4 Panels C and D).

Thereafter, each sample was assessed for protein amount by the Bradford assay (Figure 3A) and NP recovery by measuring DiI fluorescence (Figure 3B). SDS-PAGE was run to visually compare the composition of surface-adsorbed protein using different isolation techniques (Figure 3C).

**Figure 3:**
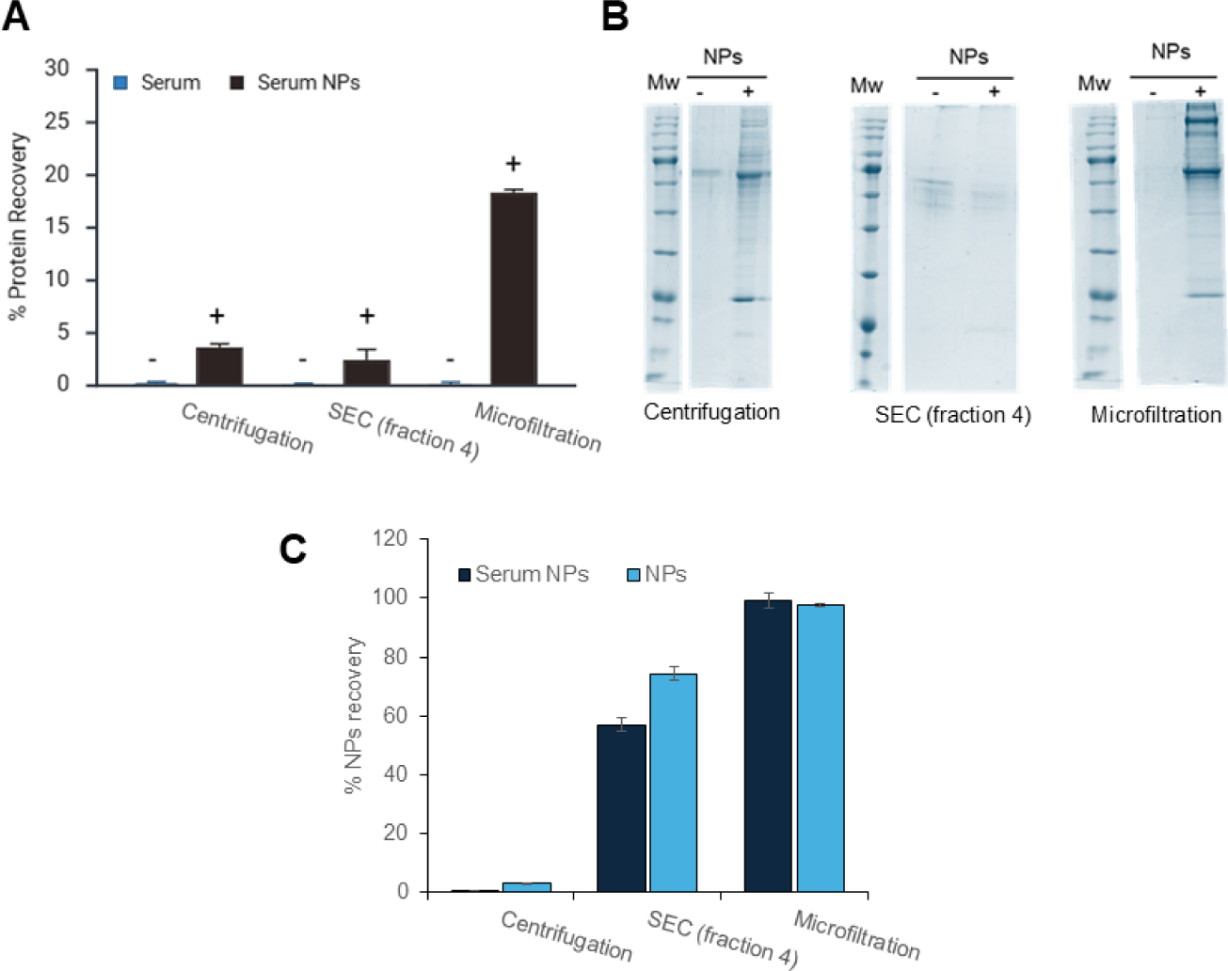
A) Protein recovery after PC purification by centrifugation, SEC, and microfiltration assessed by Bradford assays is reported in black; protein recovery of the FBS alone, used as a negative control, is in blue; the absence or presence of NPs is indicated by – and + . B) SDS-PAGE of the proteins of the PC obtained by the three separation procedures as in Panel A. For the SEC approach, fraction 4 was lyophilized before SDS-PAGE; the absence or presence of NPs is indicated by – and + . C) NP recovery assessed by DiI fluorescence analysis following the three above-mentioned separation procedures. The results of the NPs incubated with FBS are reported in black, whereas the results of the NPs alone, used as a positive control, are in blue.

The centrifugation-resuspension procedure resulted in very low recovery of NPs, as reported in the literature.^19^ On the other hand, the SEC procedure yields a diluted protein sample that requires lyophilization before SDS-PAGE, which causes a consistent loss of protein material. Ultimately, 300 kDa microfiltration proved to be fast, easy, and reproducible, yielding a reliable quantity of both adsorbed proteins and recovered PLGA NPs, as confirmed by Bradford and fluorescence assays (Figure 3A,B). As visible from SDS-PAGE, numerous protein bands were detected after the microfiltration procedure, not only at 60 kDa, which was expected to be serum albumin, but also bands matching higher and lower molecular weight species were observed. These findings suggest the formation of a multifaceted PC containing multiple and different proteins, as expected by recent literature.^12,19,27^

### Characterization of PLGA/PEG_2k_–PLGA_2k_ NPs PC after Serum Protein Adsorption

Following this, the PC of PEG30, PEG50, and PEG70 NPs was isolated by an optimized microfiltration procedure and thoroughly analyzed. PEG30, PEG50, and PEG70 NPs were incubated at a concentration of 0.5 mg/mL with 10% FBS for 1 h at 37 °C under gentle stirring.

A multiparametric measurement of variations occurring in NPs following incubation and isolation in FBS was performed.^19^ Each NP type exhibited properties that fell within the anticipated size range (approximately 100 nm in diameter). The *D*_H_ and PDI of all the samples increased somewhat after isolation and incubation, which is consistent with previous findings^19^ (Figure 4A,B). The NP surface charge became less negative for all of the NP variants tested upon FBS incubation (Figure 4C). Overall, these findings suggest that interactions between proteins and NPs take place during PC formation.

**Figure 4:**
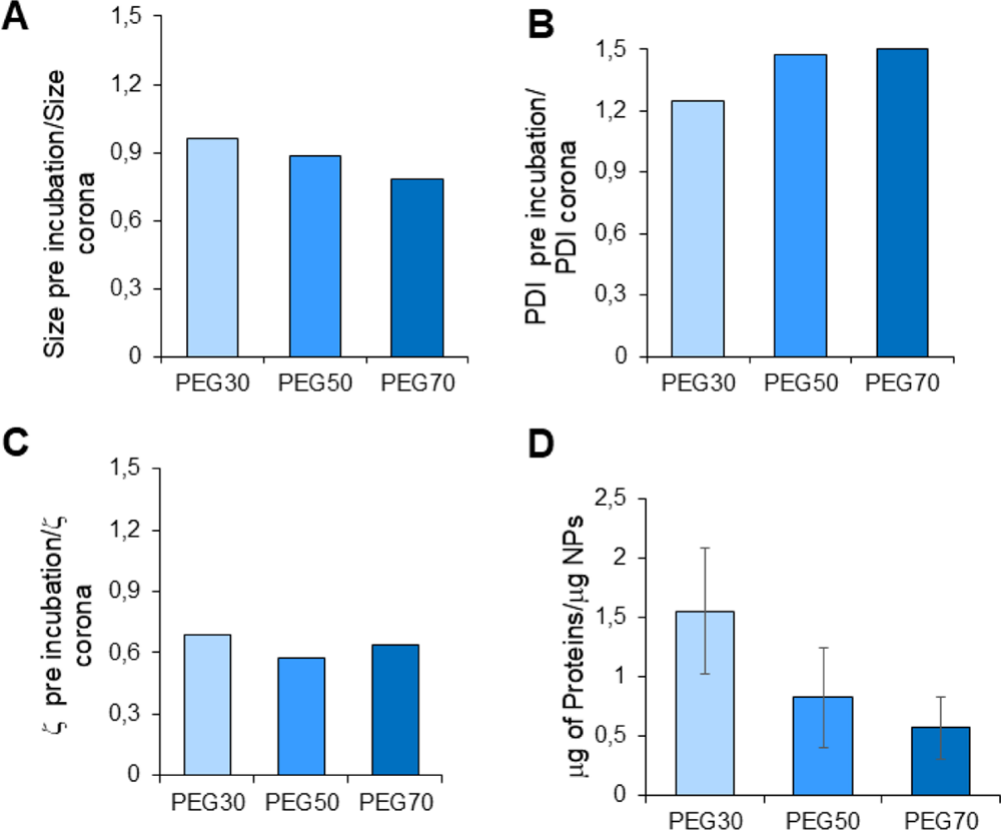
Ratio between A) Hydrodynamic diameter (*D*_H_), B) PDI, and C) surface charge (ζ) of NPs incubated with FBS before and after PC recovery. D) Amount of protein recovered for each NP sample. PC was isolated by microfiltration. The incubation of each NP type with serum was performed in triplicate.

Moreover, as reported in Figure 4D, the amount of proteins adsorbed on the NP surface drastically diminished when the PEG percentage increased. The percentage of NPs recovered did not vary for the different formulations (data not shown). As reported in the literature,^12^ the incorporation of PEG into the polymer shell reduces overall protein adsorption, with higher PEG content correlating with lower amounts of adsorbed proteins. However, rather than completely preventing protein binding, PEGylation modulates the composition and pattern of protein interactions. Therefore, a proteomics-based analysis was conducted to characterize the PC fingerprint.

### Protein Identification by NanoUPLC-MS/MS Analysis and Data Analysis

A FASP procedure was optimized to tryptically digest the adsorbed proteins,^23^ and high-resolution nano-UPLC-MS/MS was used to analyze the resulting peptides. Incubation and isolation were performed in triplicate for each NP type, and three runs were completed for each sample, thus generating nine nano-UPLC-MS/MS runs for each type of PEGylated NPs. The identification and quantification of PC were made possible by the Proteome Discoverer-based analysis of the raw data. We were able to detect a high number of proteins in the corona of the different NP systems. Therefore, the quantitative analysis of the proteins present in the three sets was carried out, and the results are reported in the heatmap in Figure S5. Most proteins were enriched on the NP surface with respect to the FBS; most of them showed the same abundance in the corona of the different NP samples, but, as clearly visible in the heatmap, there was also a consistent number of hits which are less (green) or more abundant (brilliant red).

Then, the lists of proteins were filtered to include those proteins identified and quantified in two out of three experiments and with an abundance ratio ≥3 compared to FBS to highlight the protein enrichment induced by NPs. Downstream of this process, about 300 proteins were included in three reliable lists of PC (PRIDE repository, Project Name: Proteomics of PLGA-based NPs PC after mild isolation and its impact on macrophage uptake). As shown by the Venn diagram in Figure 5 A, 65% of the proteins are common to the three PCs; 30% of the proteins are shared by two out of three coronas, and only 5% of the proteins belong individually to one NP type corona.

**Figure 5:**
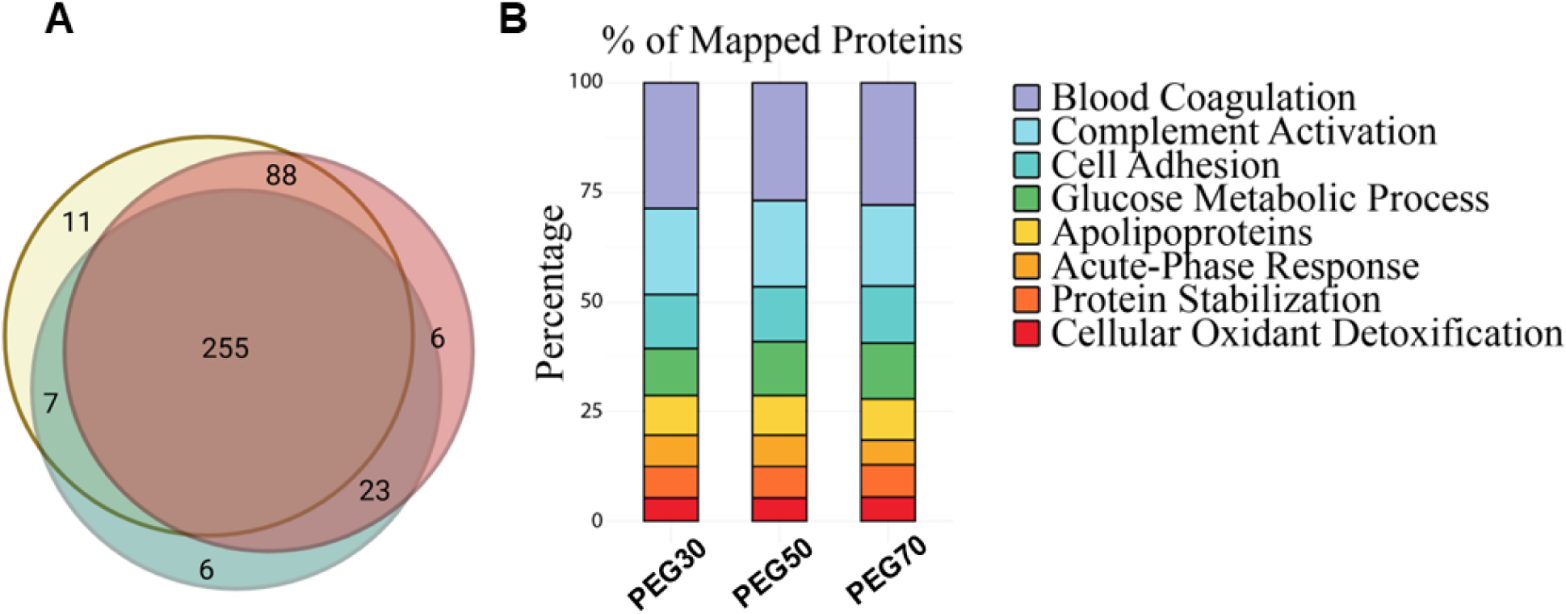
A) Venn diagram of the three lists of PC; the PC of PEG30 NPs is colored in green, of PEG50 NPs in red, and of PEG 70 NPs in yellow; B) Results of the gene ontology analysis carried out on the three lists of PC, considering the gene counts for each biological process. Scarcely populated protein pathways were excluded from the histogram.

To cluster proteins from each corona according to the biological processes and pathways in which they are involved, a gene ontology approach was employed. The histograms reported in Figure 5B, obtained using FunRich software, provide an immediate visualization of the percentages of the number of genes and therefore proteins identified in the most populated biological processes of the PC for the different types of NPs. In detail, proteins involved in the blood coagulation, complement activation, and cell adhesion pathways are widely represented in all three lists, following the literature.^15,28^

Particular attention was given to the relative abundance of specific proteins within the coronas, as this parameter is a major determinant of the biological fate of NPs. Figure 6 summarizes the distribution of proteins associated with key biological pathways across the three formulations. Notably, several proteins involved in the blood coagulation pathway were detected on the NPsurfaces (Figure 6A). Among these, three fibrinogen isoforms—α, β, and γ—were identified, with the highest enrichment observed on PEG30 NPs. Similarly, antithrombin III and vitamin K-dependent proteins were progressively less abundant as the PEG content increased, suggesting that higher PEGylation levels reduce the association of these proteins with the NPsurface.

**Figure 6:**
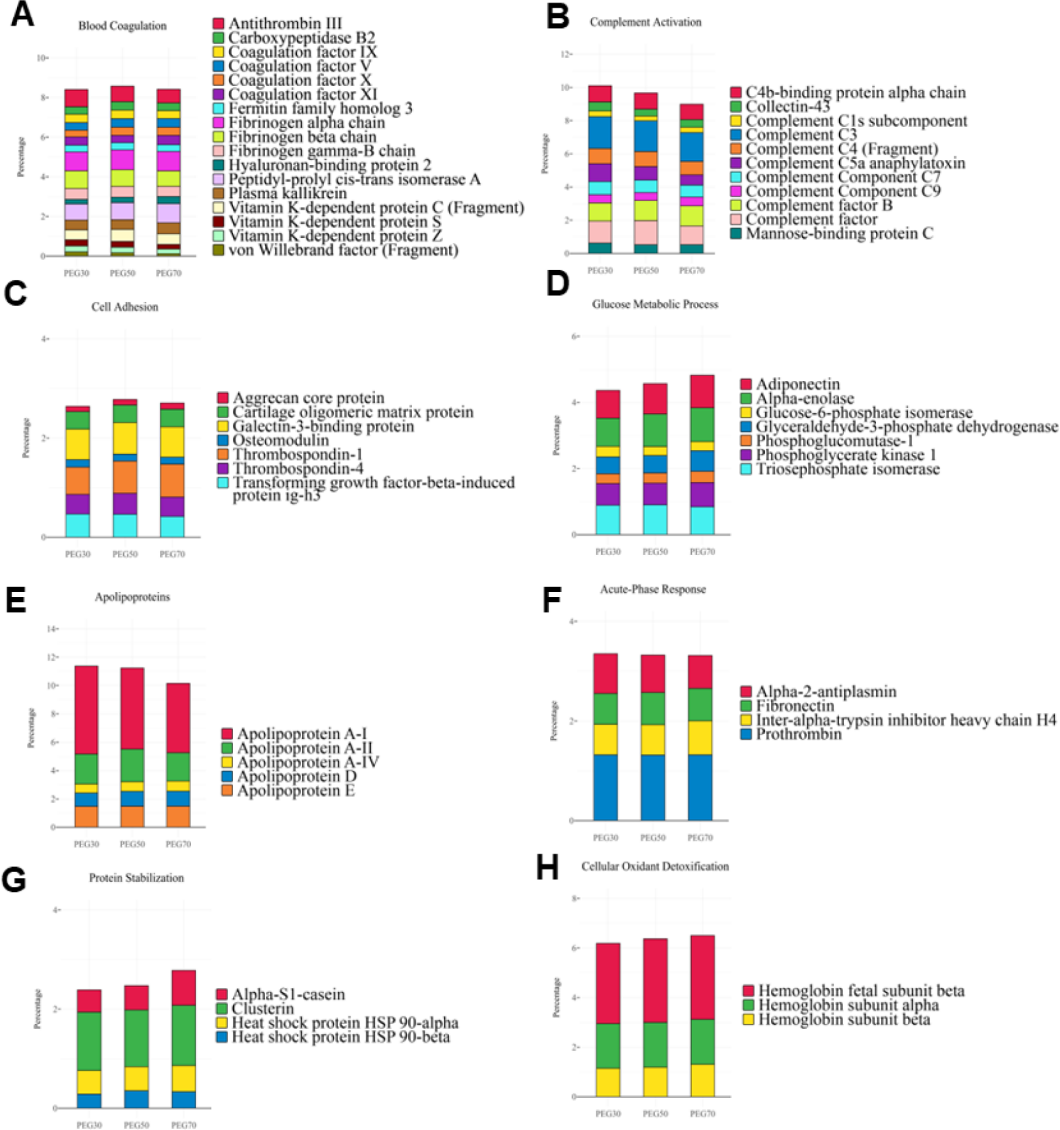
Classification of the enriched proteins belonging to PEG30, PEG50, and PEG70 NPs-PC, according to their physiological functions.

Proteins of the complement system accounted for approximately 10% of the total PC and displayed formulation-dependent trends (Figure 6B). While complement factor C3 was consistently detected across all three formulations, factors C4, C5a, and H were more abundant on PEG30 and PEG50 than on PEG70 NPs. In contrast, complement factor B showed anopposite behavior with greater enrichment on PEG70 surfaces. These findings highlight how PEGylation modulates not only the quantity but also the composition of adsorbed proteins, potentially influencing NP–immune system interactions.

Regarding those proteins belonging to the cell adhesion pathway (Figure 6C), thrombospondin-1 is more abundant on PEG50 and PEG70 than on PEG30. Among the so-called glucose metabolic process-related proteins (Figure 6D), which represent 5% of the PC, it is noteworthy that adiponectin, glyceraldehyde-3-phosphate dehydrogenase, and alpha enolase increased, in agreement with the PEG increase. Figure 6E reports the percentage of protein abundance of the apolipoproteins, which is approximately 12%. The Apolipoprotein A1, the most abundant protein in this pathway, significantly decreased as PEG increased. Prothrombin, fibronectin, and alpha-2-antiplasmin are equal and not influenced by PEGylation (Figure 6F). The most varying protein is alpha-S1-casein, which has a greater affinity for PEG70 particles (Figure 6G,H). Thus, although the qualitative composition of the PC of the different PEGylated NPs appeared similar, the quantitative analysis revealed some differences. Since many proteins considered opsonins and dysopsonins, as well as proteins involved in cell adhesion, are present in different amounts on the surface of NPs, we proceeded to measure the macrophage uptake of our PEGylated NPs to correlate the PC composition with cellular uptake.

In parallel, PLGA100-NPs underwent the same study to learn more about the function of PEG in protein recognition. Thus, as illustrated in Figure S6A, the same multiparametric measurement of NP changes after incubation and isolation in FBS was carried out. While the NP surface charge fluctuated relatively little, the D_H_ and PDI rose far more than what occurred for PEGylated NPs, as predicted.^12^ Lastly, the absence of PEG in the polymer shell raises total protein adsorption, according to published research. Thus, the identical proteomics-based analysis was carried out to describe the PC fingerprint, and the proteins were clustered according to the biological processes and pathways in which they are involved using the FunRich tool, as reported in Figure S6B. In contrast to PEGylated NPs, proteins implicated in the stabilization and cell adhesion pathways have significantly risen, but those recognized as apolipoproteins have significantly decreased and those associated with the cellular oxidation detoxification process have disappeared entirely. This clearly and predictably shows that a great variation of PC is determined by the lack of PEG.

The relative abundance of many key proteins within the PC also corroborates this observation. As illustrated in Figure S6C, the PC of PLGA100-NPs lacks the antithrombin III, complement factor B, fibrinogen α, and alpha-2-antiplasmin that were found in their PEGylated counterparts, but vitamin-K-dependent proteins, adiponectin, and apolipoprotein A1 are adhered to a far higher degree than those of PEG-bearing NPs. Thus, given the significant differences in the amount and PC composition caused by PEGylation, these results demonstrate how unprofitable it can be to directly compare the PC situation of PLGA100 with those of PEG30, PEG50, and PEG70.

### Uptake in Macrophages

We investigated the correlation between the uptake pattern into the bone marrow-derived macrophage cell line (BMDM) and the PC of NPs to relate the type of proteins and their contribution to macrophage recognition.

To quantify the cellular internalization of NPs, BMDMs were incubated with fluorescent DiI-loaded NPs (50 μg/mL) for 1, 4, and 24 h, and the fluorescence was measured in viable cells by flow cytometry (Figure S7). At 50 μg/mL, NPs did not affect cell viability up to 24 h of incubation (Figure S8). Untreated cells served as a negative control. Results demonstrated that both the percentage of fluorescently positive cells and the fluorescence intensity increased over time (Figure 7A–C). After 24 h, nearly 100% of cells were internalized the DiI-labeled formulations (Figure 7C). Among the formulations, PEG30 and PEG50 exhibited similar uptake levels at all time points, while PEG70 showed significantly earlier internalization (Figure 7A,B). Notably, lipopolysaccharide (LPS) stimulation did not alter NP uptake in BMDMs (Figure 8A–C). After 24 h of incubation, 100% of the BMDMs internalized the different NPs, and the PEG70 NPs were surprisingly internalized in greater amounts in BMDMs with and without LPS treatment (Figures 7C and 8C).

**Figure 7:**
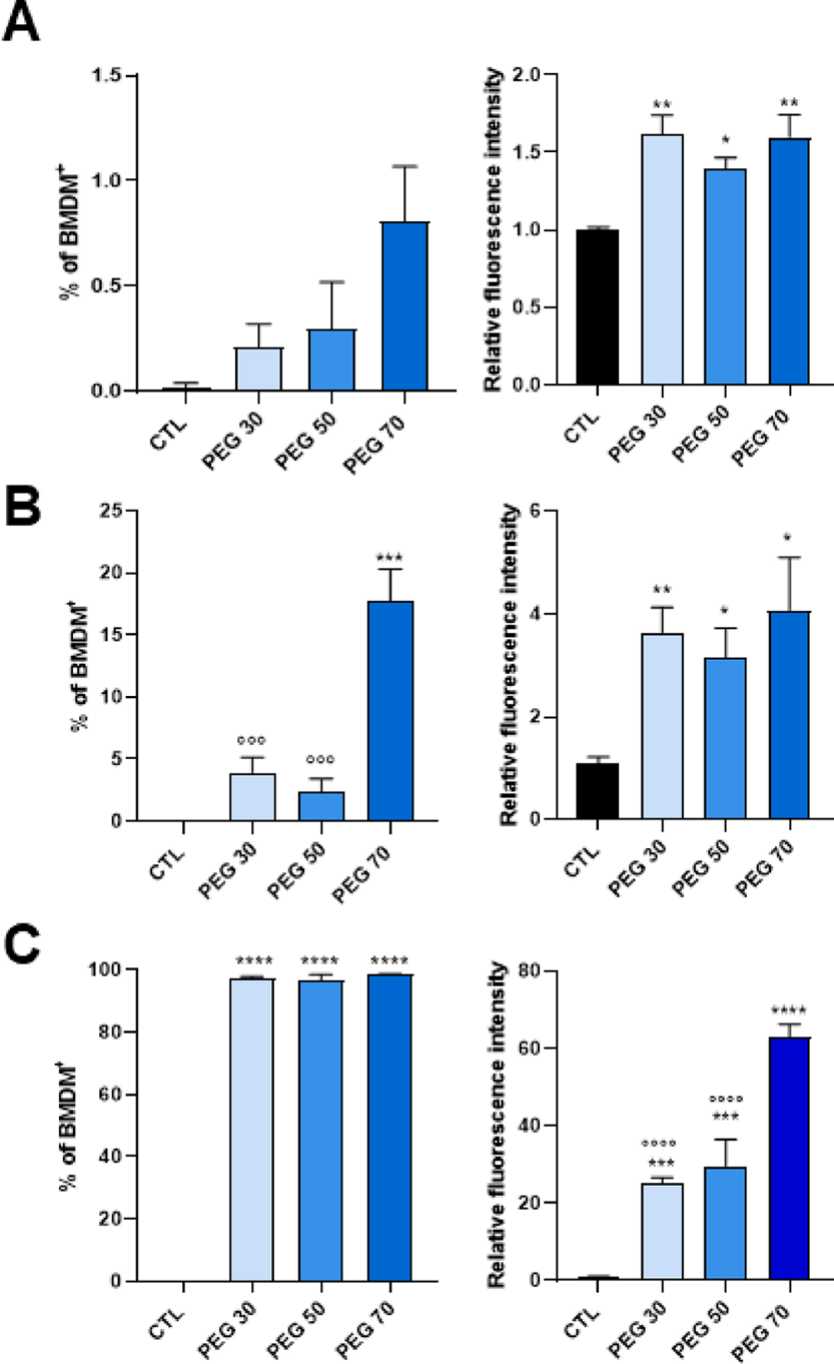
Percentage and relative fluorescence intensity of DiI-positive BMDMs analyzed by flow cytometry after treatment with DiI-loaded NPs for 1 h (A), 4 h (B), and 24 h (C). All values are normalized to untreated BMDMs. Data are expressed as mean ± S.E.M., with *N* = 2–3 independent biological replicates. Statistical significance was determined by one-way ANOVA with Dunnett’s post hoc test (*p < 0.05, **p < 0.01, ***p < 0.001, ****p < 0.0001 vs CTRL; °°°*p* < 0.001, °°°°*p* < 0.0001 vs PEG70).

**Figure 8:**
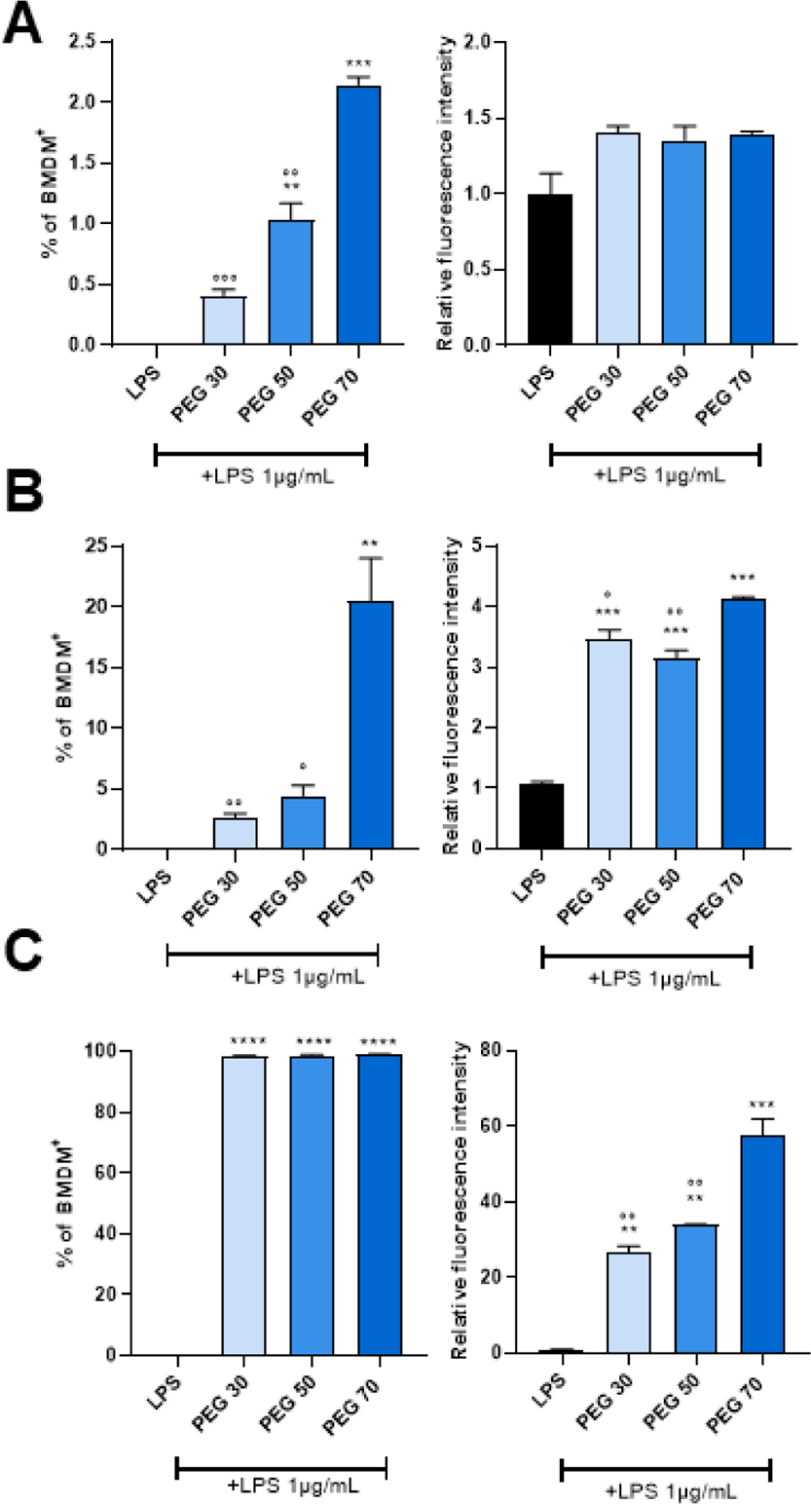
Percentage and relative fluorescence intensity of DiI-positive BMDMs analyzed by flow cytometry prestimulated with LPS (1 μg/mL, 30 min) and after treatment with DiI-loaded NPs for 1 h (A), 4 h (B), and 24 h (C). All values are normalized to untreated BMDMs. Data are expressed as mean ± S.E.M., with *N* = 2–3 independent biological replicates. Statistical significance was determined by one-way ANOVA with Dunnett’s post hoc test (*p < 0.05, **p < 0.01, ***p < 0.001, ****p < 0.0001 vs CTRL; °*p* < 0.05, °°*p* < 0.01, °°°*p* < 0.001 vs PEG70).

NPs’ cellular uptake was also visualized using confocal microscopy, as shown in Figure 9, revealing marked differences in the internalization efficiency. At earlier time points (1 and 4 h), NPs with high PEG content displayed better intracellular fluorescence; after 24 h, all the formulations showed strong intracellular signal (Figure 9A). Similar results were observed for LPS-stimulated BMDMs (Figure 9B). In general, proteins from the complement, apolipoprotein, and globulin categories showed a correlation with cellular uptake: their presence in the corona modulates the uptake of NPs in macrophages.^24^

**Figure 9:**
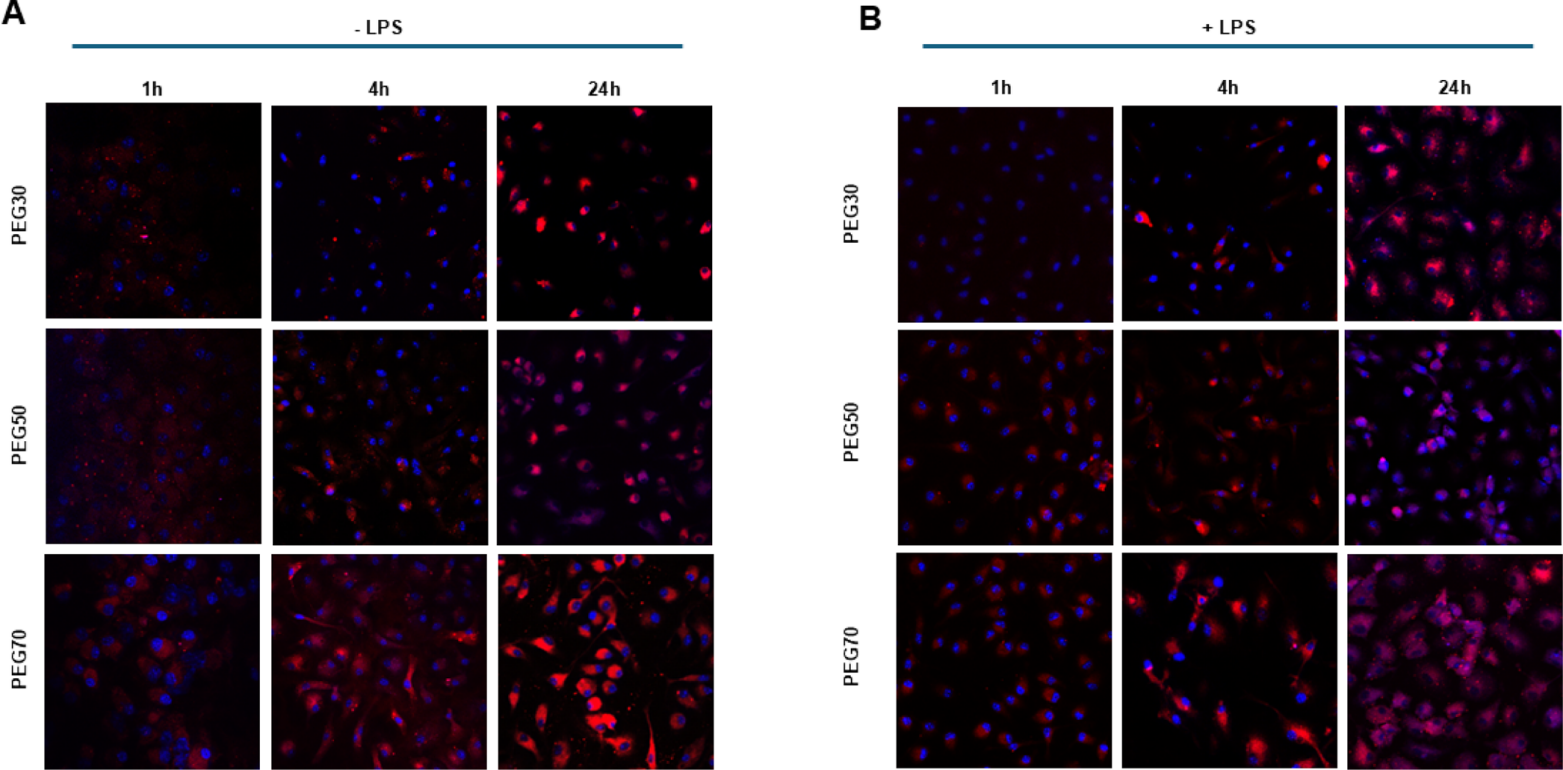
Representative confocal fluorescence images showing intracellular uptake of fluorescently labeled NPs (red) (50 μg/mL) in BMDMs not stimulated (A) or prestimulated with LPS (1 μg/mL, 30 min) (B) after 1, 4, or 24 h of incubation. Cell nuclei were counterstained with Hoechst 33342 (blue).

## Discussion and Conclusions

Understanding and precisely tuning the interactions between NPs and biological systems have become central to both nanotoxicity and nanomedicine research. Physiological environments such as blood, serum, and cellular cytoplasm contain complex protein mixtures and, when engineered NPs enter these environments, they spontaneously adsorb proteins, forming what is known as PC, a dynamic layer that can comprise hundreds of different proteins.^29^ The formation of the PC significantly alters the physicochemical properties of NPs, including their size, zeta potential, morphology, and aggregation state. In parallel, it reshapes the way NPs interact with biological systems, influencing their kinetics, transport mechanisms, and overall reactivity.^30^ These transformations are critical in determining NPs behavior in biological settings, affecting their potential applications in drug delivery, imaging, and therapeutics, as well as their possible toxicological effects.^31^ For instance, adsorbed proteins can function as opsonins, enhancing NPs uptake by phagocytic cells^31^ or as dysosponins reducing cellular recognition and prolonging NPcirculation by limiting interactions with immune components.^32^ Moreover, it is now established that the synthetic identity of NPs, determined by their material composition and surface characteristics, dictates the makeup of the PC and subsequent cellular interactions.^33^ The effect of surface chemistry on corona formation has also been extensively studied.^34^ Notably, applying coatings such as PEG can mitigate protein adsorption, helping to modulate NPs behavior in biological settings.^28^ These findings are critical for developing NPs optimized for biomedical applications, from drug delivery to imaging and diagnostics.

In this paper, we finally attempted to link systematically the chemical identity and colloidal properties of PEG–PLGA NPs, PC composition, and uptake by BMDMs. First, the PC isolation protocol based on 300 kDa microfilters was successfully optimized to recover PC in its most native form. Afterward, this protocol has been applied for the analysis of well-defined differently PEGylated PLGA-based NPs incubated with FBS and their PC has been described by proteomics. 10% FBS was selected as the model protein source since our goal was to characterize the PC in the full BMDM cell culture medium, where the NPs uptake was assessed.

The proteins obtained were also clustered using gene ontology, and their abundance has been reported by comparing NPs with three percentages of PEG. Macrophage cell uptake has been evaluated, revealing that PEG70 NPs are internalized faster and in greater quantities. The final aim was linking NPs features, PC composition, and BMDM uptake. It was expected that a higher PEG–PLGA fraction in the NPs composition results in a protein shield that limits internalization.^28^ Surprisingly, we observed the opposite behavior, with PEG70 displaying the lowest protein binding correlated with the highest rate and extent of macrophage uptake. Indeed, the qualitative character of the corona seemed to have a greater impact on BMDM absorption than the total protein content alone.

PEGylation had a dual effect: it modulated both the physicochemical properties of the NPs and the type and quantity of adsorbed proteins. The increase of PEG–PLGA in the NPs had a significant impact only on size, which was progressively decreased (140, 112, and 95 nm for PEG30, PEG50, and PEG70, respectively). Despite being prepared at different feed ratios of PEG–PLGA, the NMR-measured PEG surface amount was much lower than the designed one, being approximately half that theoretically calculated. A substantial portion of PEG chains can become entrapped within the PLGA (or other polymeric) matrix during the nanoprecipitation process. In particular, for PEG–PLGA block copolymers, a fraction of PEG chains may orient inward toward the nanoparticle core or internal interfaces, rather than projecting outward to the surface as intended.^35,36^ As a consequence, the actual PEG surface densities were similar for all NP variants (about 70 PEG chains/100 nm^2^). All of the formulations approached the threshold for dense PEG brush conformation, as supported by FALT measurements and theoretical calculations (comparable RF*/d* values of ca. 3).

BMDM uptake did not simply correlate with the total protein content but appeared to be more strongly influenced by the qualitative profile of the corona. Proteomic profiling revealed that while many proteins were commonly shared across all coronas, their relative abundances were different. It seems that the behavior observed on macrophages can be clarified by looking at the abundance of several key opsonins and dysopsonins and at the so-called opsonin/dysopsonin balance, which is critical to determine the biological fate of diverse NPs.^32^ PEG30 NPs, having the largest size and highest relative protein adsorption, were enriched in fibrinogen isoforms and components of the complement and coagulation cascades, proteins classically associated with opsonization and immune recognition.^37^ However, although less enriched in classical opsonins like fibrinogen or complement C4/C5a, PEG70 was more readily internalized by BMDMs at early and late time points. This suggests that specific proteins present in the PEG70 corona, such as complement B or α-S1-casein, may actively facilitate recognition and uptake or, alternatively, that reduced steric hindrance at the surface enables more favorable interactions with scavenger or complement receptors. On the contrary, those proteins present in the PEG30 corona, such as Apo-A1, may reduce the uptake working as a dysopsonins.^24,37,38^ Indeed, Apo-A1 has been shown to prevent macrophages, in an abundance-dependent way and with a strong positive correlation with the lifetime of NPs in the systemic circulation.^38^

Notably, lipopolysaccharide (LPS) stimulation did not significantly affect uptake patterns, indicating that the differences observed are intrinsic to the NP–protein interface rather than a result of altered macrophage activation states.

Taken together, these results demonstrate that PEGylation modulates the makeup of the PC in a nonlinear manner, converging to define the cellular uptake behavior. The higher uptake of more PEGylated NPs is, at first glance, surprising compared with the current literature but should be considered as the result of a series of experimental features that must be examined one by one. The first was the choice to use bovine serum, which is useful for mimicking what happens in cellular assays, although it is very different from human serum and plasma, in which studies like ours are often conducted. The second factor is the use of 2 kDa PEG instead of 5 kDa PEG, which induces a less pronounced stealth effect and a greater protein absorption that is reflected in macrophage uptake. On the other hand, the seemingly controversial effect of the greater internalization of PEG70 compared to PEG50 and PEG30 could be due to a combination of issues both as structural factors (size, PEG density) and as compositional factors (specific protein adsorbates). This underscores the importance of integrating surface chemistry, proteomic characterization, and cellular readouts to inform the rational design of stealth or targeted nanocarriers’. Future analysis will also be performed on human serum since the differences in protein type, amount, and function compared to the FBS can influence the PC composition.

## Conclusions

This study demonstrates that although the surface presentation of PEG on PEG–PLGA nanoparticles is comparable, the nature and composition of the protein corona formed upon incubation in fetal bovine serum differ significantly, leading to unexpected uptake by bone marrow-derived macrophages. Proteomic analysis revealed that the opsonin-to-dysopsonin balance plays a more decisive role than interactions with individual proteins. Our findings highlight that nanoparticle surface design alone is insufficient to reliably predict biological behavior. Instead, the formation of the protein corona acts as a critical intermediate layer that redefines the nanoparticle identity and profoundly influences *in vitro* performance. Therefore, a deep understanding of the protein corona is essential for optimizing the therapeutic efficacy of nanoparticle-based systems

## Supplementary Material



## Data Availability

Proteomics data are disposable on PRIDE repository, Project Name: Proteomics of PLGA-based NPs PC after mild isolation and its impact on macrophage uptake. Project accession: PXD061055.

## Notes

Technological properties of PLGA and PEG formulations; DiI entrapment analysis; ^1^H NMR spectra of PEG formulations; Additional fluorescence and Bradford analysis on PC-NPs after SEC and microfiltration; Proteomic-based heat map of PC for the three PEGylated NPs; PC analysis of PLGA100 NPs; Fluorescence images of BMDMs incubated with DiI-loaded NPs; Percentage of viability of BMDMs incubated with NPs (PDF)
