# Supplementary material for: PEGylation-Driven Remodeling of the Protein Corona on PLGA Nanoparticles: Implications for Macrophage Recognition
Source: Biomacromolecules. 2025 Oct 27;26(12):8522–34. doi: 10.1021/acs.biomac.5c01369 (PMC12690586; doi:10.1021/acs.biomac.5c01369)
Supplement: Supplementary file 1 [file bm5c01369_si_001.pdf]

Supplementary Information

PEGylation-driven remodeling of the protein corona on PLGA nanoparticles: implications for macrophage recognition

*Lucio Spinelli[a§], Pasquale D'Anna[a§], Elva Morretta[a], Chiara Cassiano[a], Virgilio Piccolo[a], Martina de Rosa[a], Rebecca Amico[a], Paola de Cicco[a], Diego Brancaccio[a], Claudia Conte[a], Angela Zampella[a], Fabiana Quaglia[a] and Maria Chiara Monti[a]**

[a]University of Napoli Federico II, Department of Pharmacy, Via D. Montesano 49, 80131, Naples, Italy

*Email: mariachiara.monti@unina.it

[§]L.S. and P.DA. contributed equally to this work

Keywords: Protein Corona, Proteomics, Serum Proteins, Polymeric Nanoparticles, Macrophages Uptake.

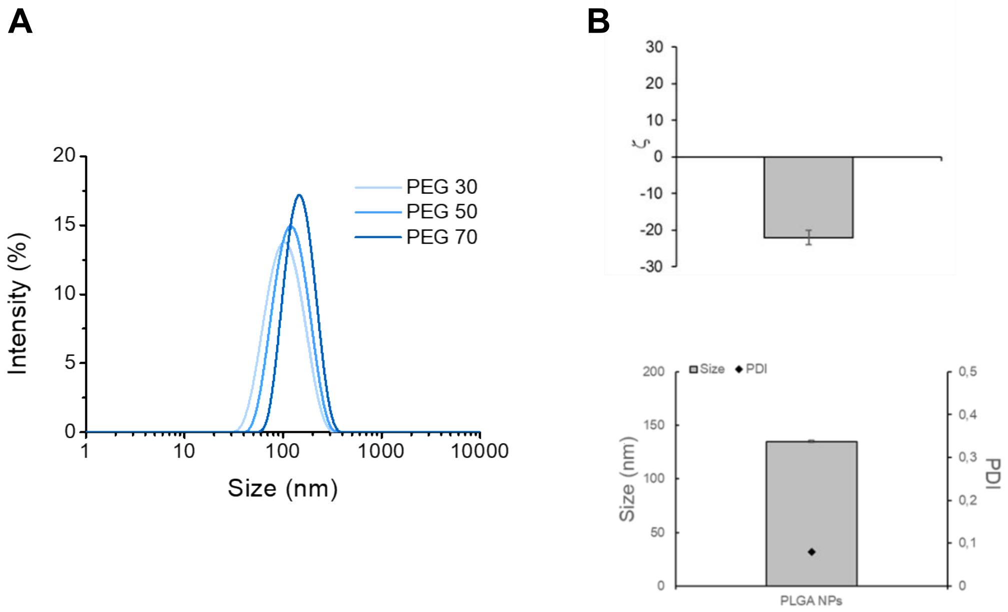

Figure S1: Panel A reports the distribution curves of PEG 30, PEG 50 and PEG 70; Panel B reports the properties of DiI-loaded PLGA NPs.

A

Calibration curve DiI in THF

Conc (µg/mL)	ABS (550 nm)
0.2	0.01
0.4	0.028
1	0.078
2	0.16
4	0.37
10	0.98

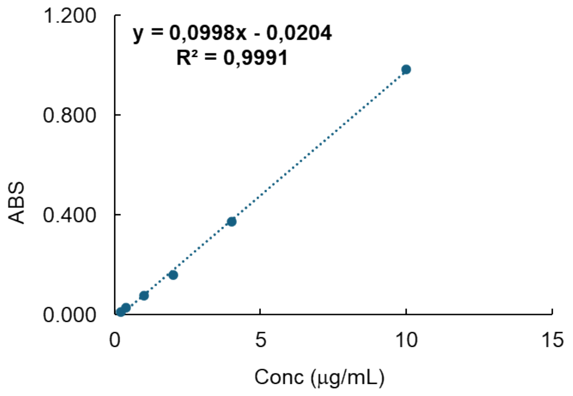

B

DiI entrapment analysis

Sample	ABS (550 nm)	µg DiI (surnatants)	% DiI free	DiI Theoretical Loading (%)	DiI Actual Loading (%)	Entrapped DiI (%)
PEG 30	0.04	0.57	4.6	0.5	0.47	95.4
PEG 50	0.11	1.3	10.3	0.5	0.44	89.7
PEG 70	0.29	3.1	25	0.5	0.37	75

Figure S2: Panel A reports the calibration curve of DiI in THF at 550 nm; Panel B reports the ABS data to calculate the entrapment efficiency of DiI inside NPs.

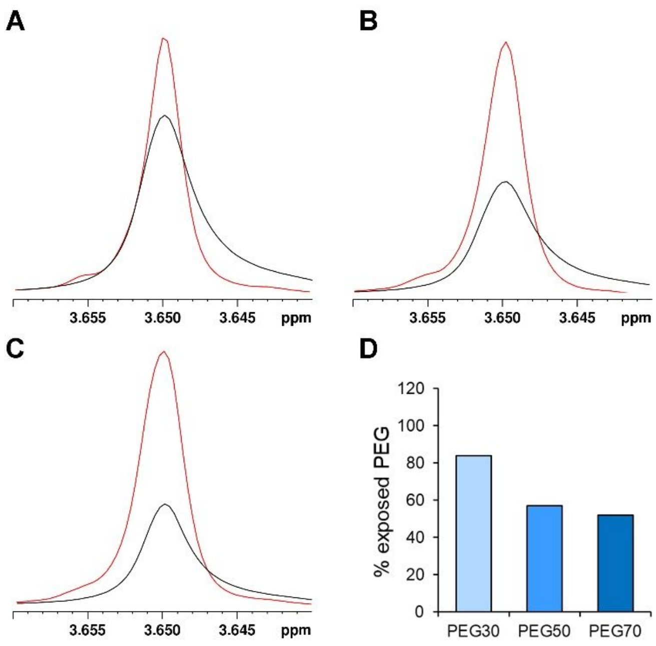

Figure S3: Overlay of 1D ^{1}H NMR spectra of PEG30 (A), PEG50 (B), and PEG70 (C) recorded in $CDCl_3$ (red) and D_2O (black). D) Percent of exposed PEG on the NPs surface as evaluated by ^{1}H-NMR.

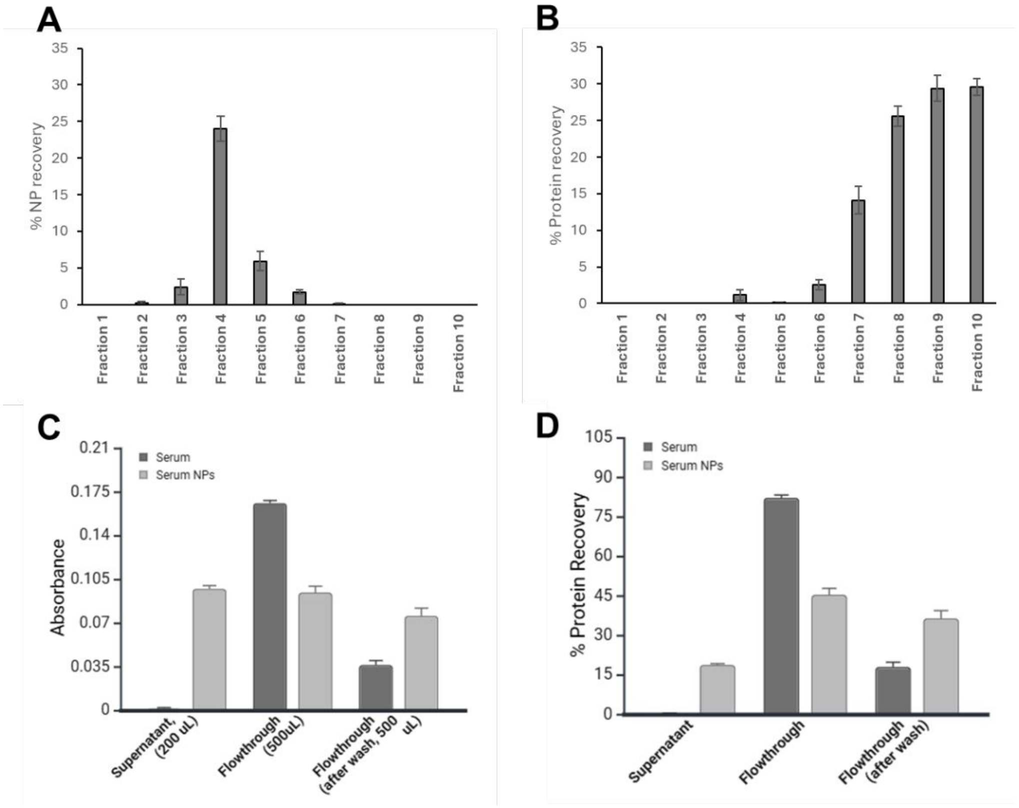

Figure S4: Panel A and B show the fluorescence and Bradford analysis of the recovery of NPs and proteins (as percentage) for each fraction eluted during SEC isolation protocol. Panel C and D show the results of the Bradford analysis (as absorbance at 280 nm and as percentage of protein recovery) of the FBS-NPs sample containing the PC and of the FBS alone as control, after microfiltration. As expected, the PC around NPs remains in the upper part of the 300 kDa microfilters (supernatant) representing the 20% proteins whereas the 100% of FBS alone pass through the microfilters to the flowthrough.

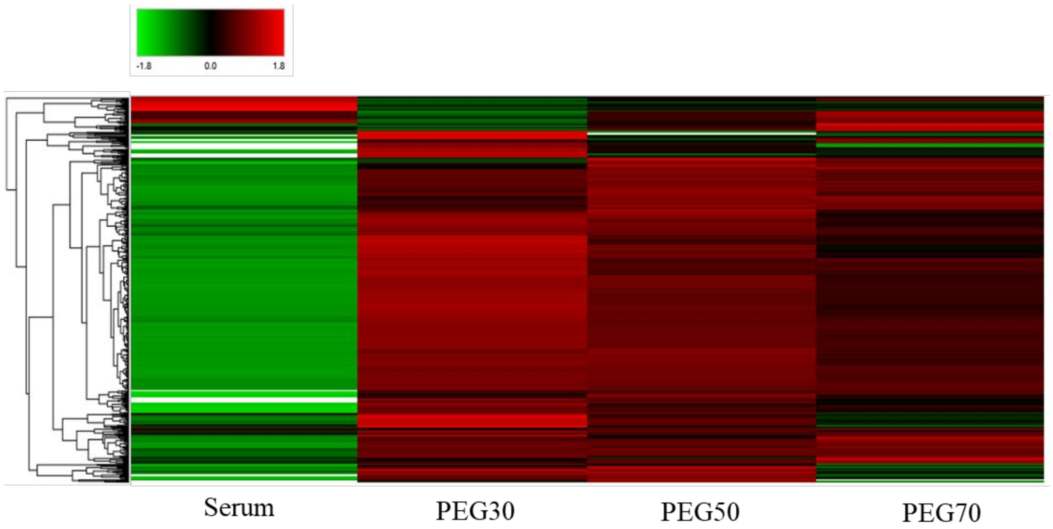

Figure S5: Hierarchical clustering of the significative alterations of the protein abundance of the PCs on the different PEGylated NPs compared to serum used as control. The red and the green colours in the heat map refer to the high and low abundance proteins, respectively.

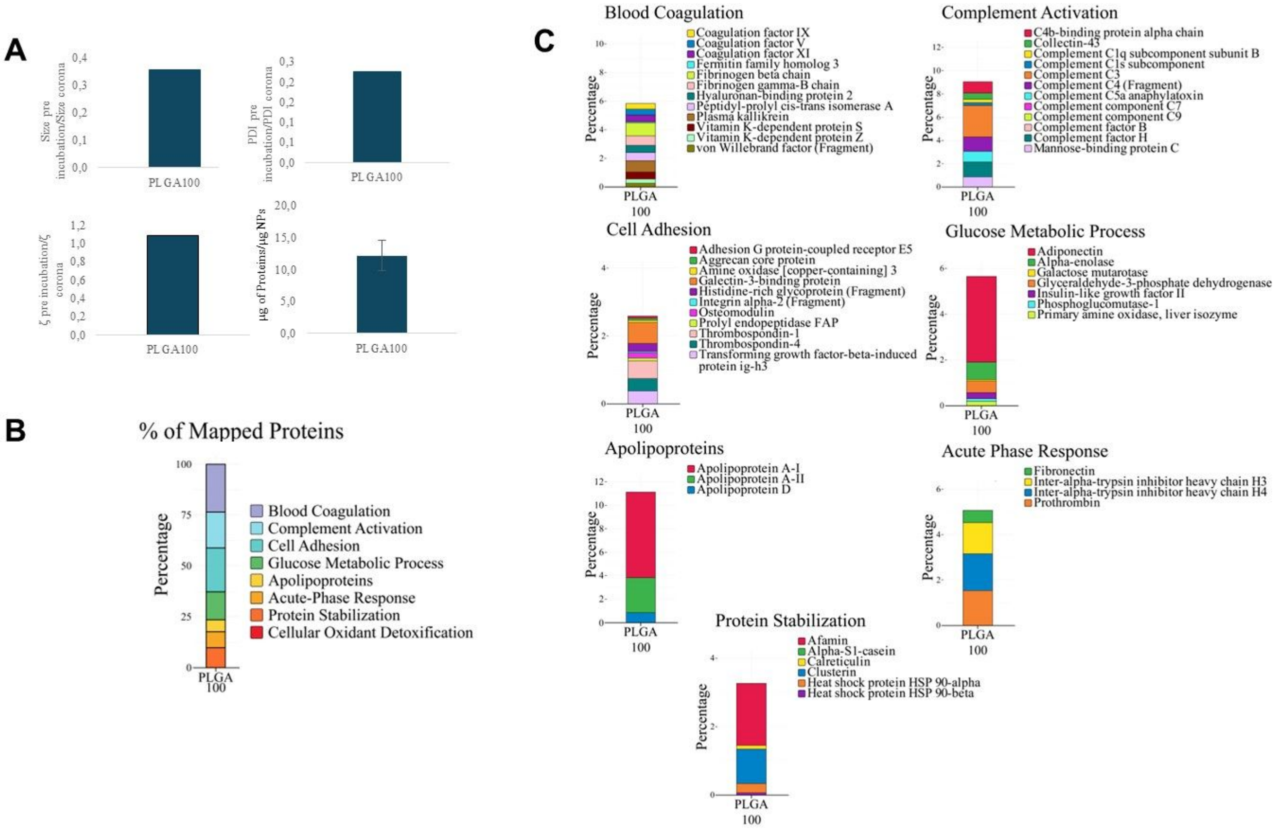

Figure S6: A) Ratio between Hydrodynamic diameter (D_H), PDI and surface charge (ζ) of PLGA100-NPs before and after incubation with FBS and recovery; amount of protein after incubation with FBS and recovery by microfiltration. B) Results of the gene ontology analysis carried out on the PLGA100-NPs-PC, considering the gene counts for each biological process. Scarcely populated protein pathways were excluded from the histogram. C) Classification of the enriched proteins belonging to PLGA100-NPs-PC, according to their physiological functions.

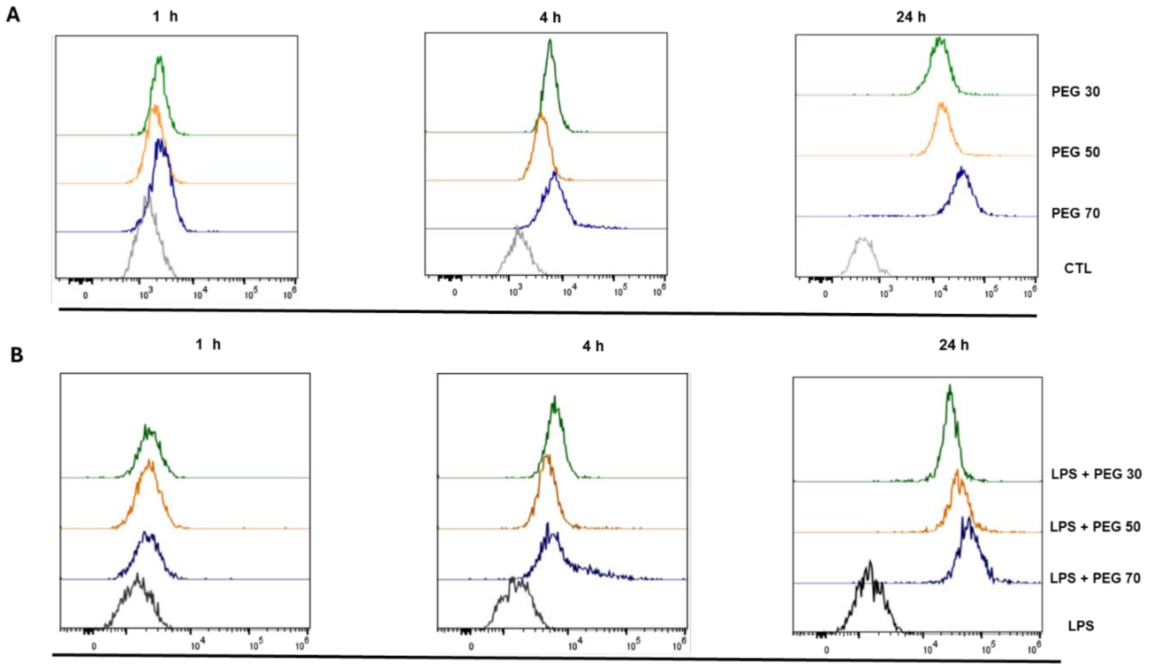

Figure S7: A) Representative fluorescence images of BMDMs incubated with DiI-loaded NP formulations (PEG 30, PEG 50 and PEG 70; 50 µg/ml) for 1, 4, and 24 hours. B) Representative fluorescence images of BMDMs pre-stimulated with LPS (1 µg/ml, 30 minutes) and subsequently incubated with DiI-loaded NP formulations (50 µg/ml) for 1, 4, and 24 hours.

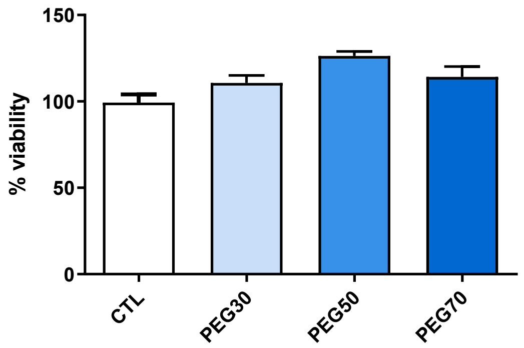

Figure S8: Viability of BMDMs incubated with NPs (50 µg/mL) for 24 h. Results are presented as percentage of live cells relative to untreated cells (CTL) (mean ± SEM; n = 3).
